# Effect of glazing technique and firing on surface roughness and flexural strength of an advanced lithium disilicate

**DOI:** 10.1007/s00784-023-05014-1

**Published:** 2023-05-13

**Authors:** Y Lu, A. M. O Dal Piva, I Nedeljkovic, J. P. M Tribst, A. J Feilzer, C. J Kleverlaan

**Affiliations:** 1 Department of Dental Materials Science, Academic Centre for Dentistry Amsterdam (ACTA), Universiteit van Amsterdam and Vrije Universiteit, The Netherlands, Gustav Mahlerlaan 3004, 1081 Amsterdam, Noord-Holland, LA Netherlands; 2 Department of Reconstructive Oral Care, Academic Centre for Dentistry Amsterdam (ACTA), Universiteit van Amsterdam and Vrije Universiteit, Amsterdam, The Netherlands, Gustav Mahlerlaan 3004, 1081 Amsterdam, Noord-Holland, LA Netherlands

**Keywords:** Lithium disilicate, Ceramic, Glass, Strength, Dental finishing, Scanning electron microscopy

## Abstract

**Objective:**

The objective of this study was to investigate the effects of glazing technique and firing on the surface roughness and flexural strength of an advanced lithium disilicate (ALD) and lithium disilicate (LD).

**Methods:**

Eight groups of bar-shaped specimens (1 mm × 1 mm × 12 mm, *N*=160, 20/group) were manufactured from ALD (CEREC Tessera, Dentsply Sirona) and LD (IPS e.max CAD, Ivoclar). The specimens were then submitted to various posttreatments: crystallization (*c*), crystallization followed by a second firing (*c-r*), crystallization with glaze in one step (*cg*), and crystallization followed by a glaze layer firing (*c-g*). Surface roughness was measured by means of a profilometer, and flexural strength was determined using a three-point bending test. Surface morphology, fractography, and crack healing analysis were conducted using scanning electron microscopy.

**Results:**

Refiring (*c-r*) did not affect the surface roughness (Ra) while applying glaze at both *cg* and *c-g* procedures increased the roughness. ALD*c-g* (442.3 ± 92.5 MPa) promoted higher strength than ALD*cg* (282.1 ± 64.4 MPa), whereas LD*cg* (402.9 ± 78.4 MPa) was stronger than LD*c-g* (255.5 ± 68.7 MPa). Refiring completely closed the crack in ALD, but it had a limited effect on LD.

**Conclusions:**

Two-step crystallization and glazing improved ALD strength compared to the one-step protocol. Refiring and one-step glazing do not increase LD’s strength, while two-step glazing has a negative effect.

**Clinical relevance:**

Besides both materials being lithium-disilicate glass ceramics, the glazing technique and firing protocol affected their roughness and flexural strength differently. A two-step crystallization and glazing should be the first choice for ALD, while for LD, glazing is optional and when necessary, should be applied in one-step.

## Introduction

In recent decades, lithium disilicate glass ceramics (LD) have become essential materials in the field of esthetic and prosthetic dentistry [1–5]. This ceramic provides a balance of strength and esthetics, which allows it to be used in wide indications, including veneers, inlays/onlays, crowns, and bridges for anterior and posterior teeth [6]. Due to its reasonable translucency, porcelain veneering is no longer necessary for LD restorations. Such monolithic restorations reduce the number of manufacturing procedures and eliminate the weak interface between substrate and veneering ceramic, therefore ensuring better mechanical properties and longevity [7, 8].

LD ceramic restorations can be produced by either conventional heat-pressing or computer-aided design and computer-aided manufacturing (CAD/CAM) [9, 10]. The CAD/CAM facility, also known as the milling procedure, includes hard and soft machining; owing to the desirable properties of LD [11], both machining methods are available, making it an ideal chairside restorative material. Hard machining involves milling objects from a fully crystallized ceramic block, which is time-consuming due to the hardness and wear of the milling bur [12]. Soft machining makes it easier to mill an object from a partially crystallized stage, but subsequent thermal processing is needed to achieve full crystallization [13], which is also time-consuming. An advanced lithium disilicate (ALD) was therefore developed to shorten the firing time and the whole fabrication period. “Advanced” nomenclature is not a classification of the material, but named according to the manufacturer due to its different composition from conventional LD, since ALD contains the natural mineral virgilite, which is claimed to have a synergistic effect with the lithium disilicate to improve the mechanical and optical properties of ceramics. Though it requires a slightly longer milling time, only a fast-firing cycle with glaze (4 min 30s) is needed, and such glaze firing is compulsory for matrix and glaze firing [14].

Conventionally, thermal treatment procedures for ceramic restorations are separate steps that consist of crystallization and glaze firing. However, this subsequent glazing has been reported to weaken the biaxial flexural strength of the conventional LD [15]. Recently, one-step firing combining crystallization and glazing has become an alternative to reduce the number of firings. Although it still reduces the biaxial flexural strength of as-crystallized ceramic, it still presents better initial biaxial flexural strength and fatigue strength compared to two-step firing [16]. For ALD, the literature regarding the benefits of glazing in one- or two-step procedure on the mechanical properties of ALD is still unknown.

One of the claims from the manufacturer is that the glaze firing together with the crystallization is necessary to achieve its final strength and to close some defects during the manufacturing procedure. However, the mechanism of strength improvement and repair is not clear. The glazing process consists of glaze material application and firing, while thermal treatment was reported to lead to crack healing [17, 18], and improvement of flexural strength [18]. Thus, it raises the question of whether the mechanical properties of ALD can be promoted by refiring, glaze material, or both. Therefore, this study was aimed at investigating the effects of the number of firings and glaze on the surface roughness and uniaxial flexural strength of ALD in comparison with LD. The null hypotheses consisted that (1) glazing and refiring would not influence the surface roughness of ALD and LD; (2) glazing together with crystallization (one-step) or separated glaze firing (two-step) would not promote different flexural strength values for both ALD and LD; and (3) refiring or the use of glaze material would not affect the flexural strength of ALD and LD.

## Materials and methods

### Specimens’ preparation

One hundred and sixty (*N*=160) bar-shaped specimens (1 mm × 1 mm × 12 mm) [19] were cut from the advanced lithium disilicate glass-ceramic (CEREC Tessera; Dentsply Sirona, Hanau-Wolfgang, Germany—ALD) and precrystallized lithium disilicate glass-ceramic (IPS e.max CAD; Ivoclar, Schaan, Liechtenstein—LD) using a diamond-coated saw in a precision cutting machine (Isomet 1000; Buehler, Lake Bluff, IL) under water cooling. All specimens were polished until the final dimension using SiC abrasive papers (Ecomet; Buehler Ltd., Evanston, IL) with a grit size of P1200. After, they were cleaned in an ultrasonic bath with isopropyl alcohol for 5 minutes. The materials’ information is summarized in Table 1. Note that the mineral virgilite refers to the aluminosilicate γ-LiAlSi_2_O_6_, but a recent study concluded that the crystal structure in ALD is not in the range of mol% SiO_2_ of virgilite definition [20].

**Table 1:** Materials’ name compositions and batch numbers

Material	Commercial name & manufacturer	Composition (wt%)	Batch number
Advanced lithium disilicate (ALD)	CEREC Tessera, Dentsply Sirona, Hanau-Wolfgang, Germany	Glass zirconia matrix, lithium disilicate, virgilite (LiAlSiO_6_)	16011535
Spray glaze	Universal Spray Glaze, Dentsply Sirona, Hanau-Wolfgang, Germany	Silicate glass, isopropyl alcohol, isobutane, propellant	L0201
Precrystallized lithium disilicate glass-ceramic (LD)	IPS e.max CAD, Ivoclar, Schaan, Lichtenstein	SiO_2_, 57.0–80.0%; Li_2_O, 11.0–19.0%; K_2_O, 0.0–13.0%; P_2_O_5_, 0.0–11.0%; other oxides	Z03B1G
Spray glaze	IPS e.max CAD Crystall glaze spray, Ivoclar, Schaan, Lichtenstein	Glazing powder, propellant, isobutene	ZL0BGT

Afterwards, the specimens of each ceramic were randomly divided into four subgroups according to the firing and glazing protocols summarized in Table 2: crystallized (*c*), crystallized and refired (*c-r*), crystallized with glaze in one step (*cg*), and crystallized followed by glaze firing (*c-g*). The application of glaze materials was conducted according to the manufacturers’ instructions. For the group *cg* with crystallization together with glaze firing, the glaze indicated from each manufacturer was used, while the Universal Spray Glaze was standardized for both ceramics only for *c-*g groups. All the firing procedures were processed in the same furnace (Programat P100; Ivoclar Vivadent, Schaan, Liechtenstein).

**Table 2:** Firing and glaze protocols used in the present study

Group	Ceramic	Firing protocol	Firing 1	Firing 2
LD*c*	Lithium disilicate (LD)	*c*: crystallization	LD crystallization firing:* Closing time: 6 min. Stand-by temperature: 403 °C. Heating rate: 60 °C/min. Firing temperature: 770 °C. Holding time: 10 s. Heating rate: 30 °C/min. Firing temperature: 850 °C. Holding time: 10 min. Vacuum 1: 550 until 770 °C. Vacuum 2: 770 until 850 °C. Long-term cooling: 700 °C/min	–
LD*c-r*		*c-r*: crystallization + refiring		*Universal Spray Glaze firing*
LD*cg*		*cg*: one-step crystallization and glaze firing		–
LD*c-g*		*c-g*: two-step crystallization and glaze firing		*Universal Spray Glaze firing*
ALD*c*	Advanced lithium disilicate (ALD)	*c*: crystallization	ALD crystallization firing:** Closing time: 2 min. Pre-heating temperature: 400 °C. Heating rate: 55 °C/min. Firing temperature: 760 °C. Holding time: 2 min. Vacuum 1 and 2: off. Long-term cooling: 0 °C/min	–
ALD*c-r*		*c-r*: crystallization + refiring		*Universal Spray Glaze firing*
ALD*cg*		*cg*: one-step crystallization and glaze firing		–
ALD*c-g*		*c-g*: two-step crystallization and glaze firing		*Universal Spray Glaze firing*

*LD crystallization firing is the same protocol indicated for the firing of IPS e.max CAD with application of IPS e.max Crystall Glaze Spray in one-step; **ALD crystallization firing is the same protocol indicated for matrix firing, one-step crystallization/glazing firing and Universal Spray Glaze firing

### Surface roughness (SR)

For each group, two specimens were produced (14 mm × 12 mm × 1 mm) to evaluate their surface roughness using a profilometer (SJ-400, Mitutoyo Corporation, Japan). Before the examination, the specimens were cleaned with isopropyl alcohol in an ultrasonic bath for 5 minutes. Five measurements were performed on each specimen in five different areas (read length: 7.5 mm, speed: 0.2 mm/s). The analysis was performed with ISO 4287-1997 standard, Gaussian filter, and a cut-off wavelength value of 2.5 mm. The average values for average roughness (Ra), the vertical distance from peaks to valleys (Rz), and the space between defects (RSm) were recorded (in μm) for each group.

### Flexural strength (FS)

Twenty specimens (*n*=20) from each group were examined in a 3-point bending test using a universal testing machine (Instron 6022; Instron Limited, High Wycombe, UK). The dimensions of each specimen were checked (1 × 1 × 12 ± 0.2 mm) using a digital caliper (Absolute 500-196-20, Mitutoyo, Takatsu-ku, Japan) before the bending test. The test was conducted using a ball-in-hole device [19] consisting of a metal base with a hole (10.1 mm diameter) and a stainless steel ball (10 mm diameter). The specimens were placed in the internal space of the base and stabilized by two supports 10 mm apart. The load was applied in the middle of the surface using a load cell (1000 N) with a crosshead speed of 0.5mm/min. Flexural strength (MPa) was calculated according to the following equation based on ISO 6872-2015:

$${\sigma}_u=\frac{3 Pl}{2b{h}^2}$$

where *P* is the maximum load (in N), *l* is the distance between the two supports (in mm), *b* represents the specimen width (in mm), and *h* is the specimen thickness (in mm).

### Scanning electron microscopy (SEM)

The fractured pieces from the FS test were collected for failure analysis. They were first checked under an optical microscope to identify the possible location of fracture origins. Then, representative specimens from each group were gold-coated and observed under an SEM (EVO LS15; Carl Zeiss, Oberkochen, Germany) to identify the fracture features. In addition, specimens from the roughness test from each group were also examined under SEM at 1000x magnification to examine the surface topography. To understand how the refiring influences strength, a crack healing analysis was conducted. For that, an indentation was made by a Vickers indenter (HM-124 Hardness Testing Machine, Mitutoyo Corp., Kanagawa, Japan) on the polished surfaces of crystallized ALD and LD at a load of 20N. One diagonal crack for each material was selected for SEM observation after gold coating. After the first examination, the specimens were immersed in acetone, followed by isopropyl alcohol in the ultrasonic cleaning machine to remove the gold [17]. After a refiring procedure like group *c-r*, the length of the selected crack was again evaluated at the same magnification.

### Data analysis

After a normality test, the data from FS and SR were submitted to 1-way and 2-way analyses of variance (ANOVA). After, Tukey test (*α* = 5%) was used to compare the mean values within each material and between all groups and to evaluate the interaction of the factors including material and protocol using SPSS software (SPSS Statistics 27, IBM, Armonk, New York, USA).

## Results

### Surface roughness

The mean and standard deviation of surface parameters including Ra, Rz, and RSm were exhibited in Table 3. Two-way ANOVA showed a statistically significant effect of material on Ra and Rz (Ra: *p* < 0.001, *F* = 244.89; Rz: *p* < 0.001, *F* = 60.08) while the protocol affected all SR parameters (Ra: *p* < 0.001, *F* = 75.86; Rz: *p* < 0.001, *F* = 23.42; RSm: *p* < 0.001, *F* = 15.72). ANOVA also revealed the influence of the interaction Material*Protocol for all parameters (Ra: *p* < 0.001, *F* = 75.144; Rz: *p* < 0.001, *F* = 25.391; RSm: *p* < 0.001, *F* = 27.176). LD showed rougher surface than ALD for all protocols, and the glazed protocols (*c* and *c-g*) had higher SR for both materials.

**Table 3:** Mean ± standard deviation of surface roughness (Ra, Rz, and RSm) and flexural strength for both ALD and LD according to the posttreatment protocols

Group	Ra (μm)	Rz (μm)	RSm (μm)	Flexural strength (MPa)
LD*c*	0.22 ± 0.05 ^BCb^	1.45 ± 0.59 ^Bb^	1030.2 ± 313.7 ^Aa^	358.2 ± 73.6 ^BCa^
LD*c-r*	0.17 ± 0.05 ^Cb^	1.23 ± 0.35 ^Bb^	572.3 ± 200.4 ^CDb^	370.6 ± 59.3 ^BCa^
LD*cg*	0.53 ± 0.06 ^Aa^	3.14 ± 0.42 ^Aa^	418.0 ± 59.6 ^CDb^	402.9 ± 78.4 ^ABa^
LD*c-g*	0.54 ± 0.12 ^Aa^	3.49 ± 0.94 ^Aa^	623.9 ± 139.2 ^BCb^	255.5 ± 68.7 ^DEb^
ALD*c*	0.07 ± 0.01 ^Db^	1.46 ± 0.64 ^Ba^	362.8 ± 156.5 ^Db^	195.9 ± 44.8 ^Ec^
ALD*c-r*	0.08 ± 0.02 ^Db^	1.45 ± 0.34 ^Ba^	366.9 ± 142.3 ^CDb^	313.6 ± 52.5 ^CDb^
ALD*cg*	0.25 ± 0.03 ^BCa^	1.42 ± 0.26 ^Ba^	858.5 ± 230.9 ^ABa^	282.1 ± 64.4 ^Db^
ALD*c-g*	0.26 ± 0.04 ^Ba^	1.36 ± 0.25 ^Ba^	896.7 ± 176.4 ^ABa^	442.3 ± 92.5 ^Aa^

Identical upper-case letter indicates no significant difference between all groups; identical lower-case letter means no significant difference within each ceramic

For LD, the protocol had effects on Ra (*p* < 0.001; *F* = 68.812), Rz (*p* < 0.001; *F* = 34.714), and RSm (*p* < 0.001; *F* = 16.848). Though the second firing decreased RSm, it did not affect Ra and Rz. Therefore, *c* was the posttreatment with more spaced defects. For ALD, one-way ANOVA also showed that the protocol affected Ra (*p* < 0.001; *F* = 117.10) and RSm (*p* < 0.001; *F* = 27.21), but no difference was found for Rz (*p* = 0.945; *F* = 0.125). A second firing (refiring) did not show different SR from *c*. However, the presence of glaze spray for both one-step (*cg*) and two-step (*c-g*) procedures increased the average roughness (Ra) as well as the space between defects (RSm).

### Flexural strength

The average FS and standard deviation of each group are presented in Table 3. Two-way ANOVA demonstrated a significant effect of material (*p* = 0.001; *F* = 12.61), protocols (*p* < 0.001; *F* = 9.84), and interaction Material*Protocol on the FS (*p* < 0.001; *F* = 52.407). For both ceramics, one-way ANOVA revealed that the protocol influenced on the FS (LD: *p* < 0.001; *F* = 16.415; ALD: *p* < 0.001; *F* = 47.81).

For LD, *c*, *c-r* and *cg* had similar and higher FS compared to *c-g*. However, for ALD, protocol *c-g* promoted the highest strength, which was more than twice the weakest group, *c*. Groups *c-r* and *cg* presented an intermediary behavior. Comparing all groups, the strongest groups for ALD (*c-g*) and LD (*cg*) had similar strength.

### Scanning electron microscopy

**Fig. 1:**
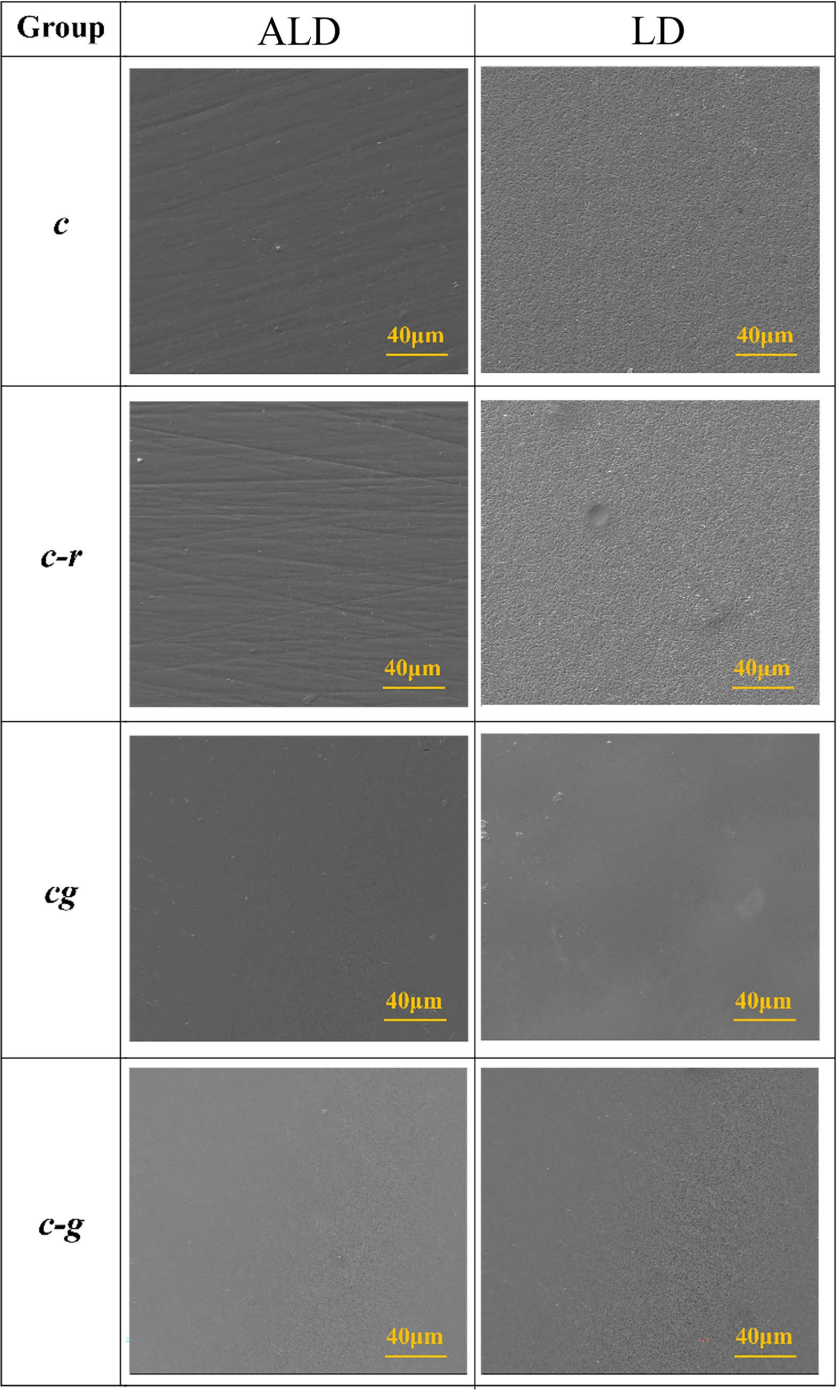
SEM images (×1000) of representative surface morphology of advanced lithium disilicate (ALD) and lithium disilicate (LD) according to different posttreatment protocols: crystallized (*c*), crystallized and refired (*c-r*), crystallized with glaze in one step (*cg*), and crystallized followed by glaze firing (*c-g*). The two ceramics showed different as-crystallized surfaces. Refiring did not affect the surface morphology of both ceramics, while glazing generated a visually silky and smooth surface

SEM images of two specimens per group are presented in Fig. 1. Both ceramics showed different as-crystallized surfaces: ALD had smoother surface but the scratches introduced by polishing remained, while LD showed rougher surface but had no scratches. Refiring did not change the surface morphology of both ceramics. Glazing generated a visually silky and smooth surface despite increased roughness parameters. One-step and two-step glazing procedures promoted similar surface for ALD. However, one-step glazing generated rougher surface than two-step for LD.

**Fig. 2:**
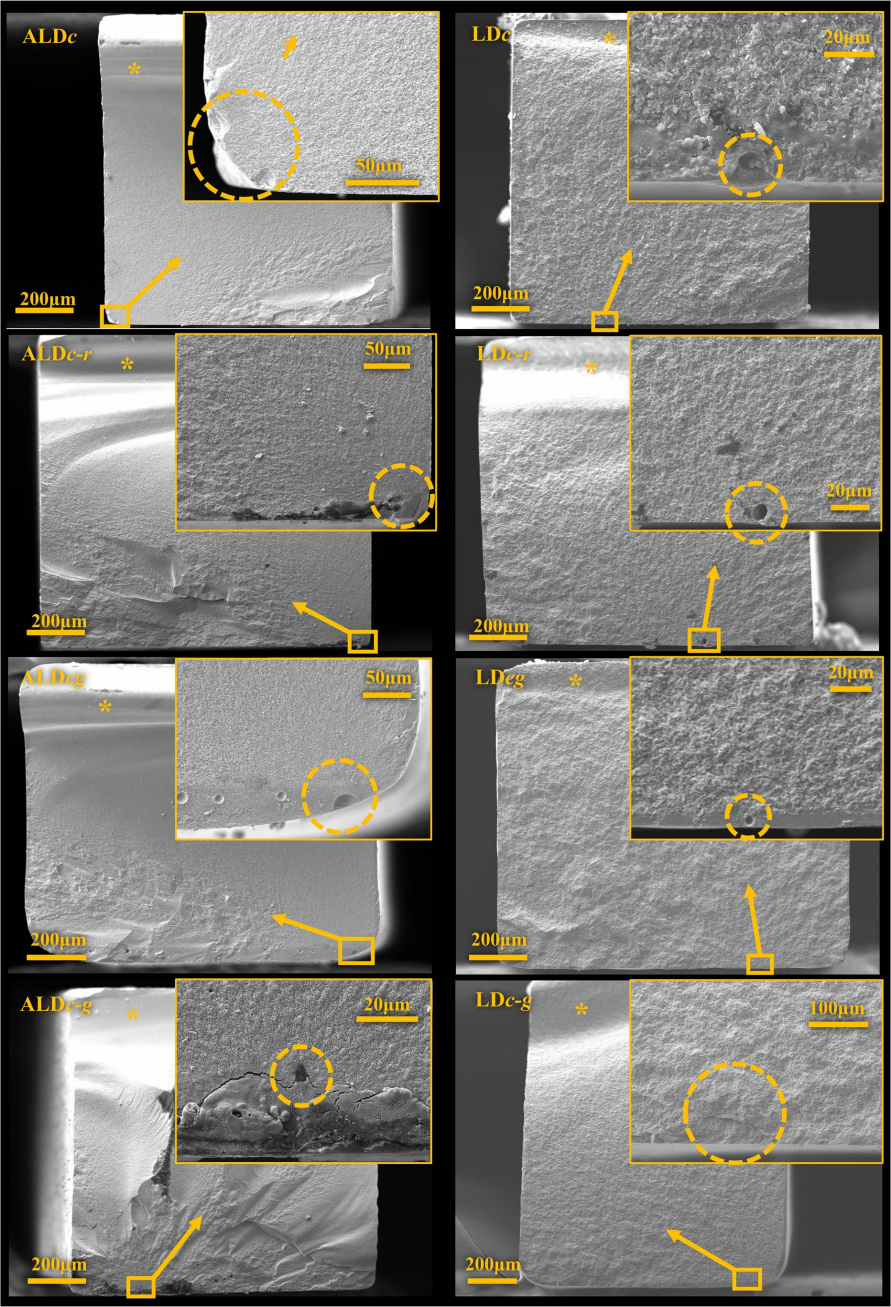
SEM images of the fractured surfaces according to the ceramic materials (advanced lithium disilicate (ALD) and lithium disilicate (LD)). The squares indicate the location of possible fracture origins; the arrows point in the directions of fracture growth; the asterisks indicate the compression curl; enlarged views of possible fracture origins are shown in the upper right corner, and the defects are marked by a dashed circle.

Fractography analysis is presented in Fig. 2. For all specimens, the fracture originated from the tensile side, as observed by the presence of compression curl on the opposite side. For both ceramics, primary or secondary fracture origins were caused by edge damages or pores within the ceramic or glaze layer.

**Fig. 3:**
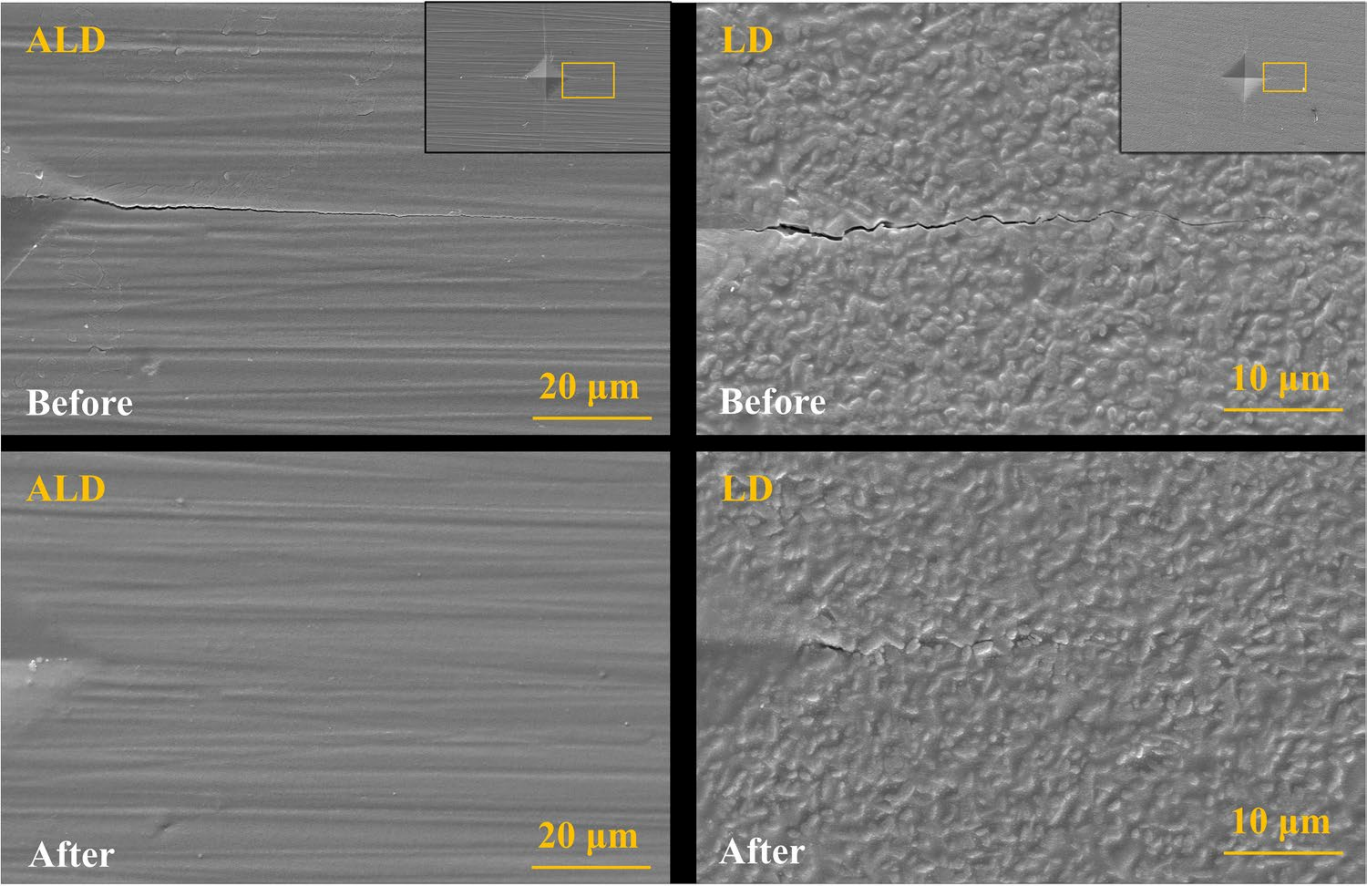
SEM images of the crack healing analysis in advanced lithium disilicate (ALD) and lithium disilicate (LD) before and after refiring. Refiring completely closed the crack in ALD, but it had a limited effect on the crack healing of LD.

The introduced cracks in crystallized ALD and LD before and after the refiring are shown in Fig. 3. The crack in ALD was completely healed, while the crack in LD was only partially closed.

## Discussion

This study was aimed at determining the influence of the number of firings and the presence of glaze layer on the surface roughness and flexural strength of two lithium disilicate-based ceramics. According to the results, the first hypothesis that glazing and refiring would not influence the surface roughness of ALD and LD was partially rejected since the glaze presence had a negative impact on the mean surface roughness. The purpose of glazing during restoration manufacturing is to improve surface quality and generate a glossy outer layer. However, the glazed surface has a greater tendency for bacterial attachment and oral biofilm formation [21] if the required threshold of 0.2 μm of surface roughness is not respected [22]. However, it is worth mentioning that other interactions can affect the microorganism adhesion, such as surface free energy and communication between existing microorganisms [21]. In this study, both ALD *cg* and *c-g* had values close to this standard, while the glazed LD groups showed rougher surfaces. This could be attributed to the surface properties of LD as well as handling. For both materials, the average roughness after glazing was higher compared to *c* groups. This result is consistent with a previous study [23] in which glazed lithium disilicate presented a rougher surface than polished. In addition, the roughness of one- and two-step glazed ALD was similar, revealing that the glazing protocols do not influence the surface quality of ALD. This was also confirmed by SEM images since ALD*cg* and ALD*c-g* showed similar surface morphology. For LD, refiring did not influence the average surface roughness (Ra), but it seems that LD*cg* and LD*c-g* have different surface morphology according to SEM images. This could be because two different glaze materials used for LD in different glazing protocols have different properties.

The second hypothesis that glazing together with crystallization (one-step) or separated glaze firing (two-step) would not promote different strength values of both ALD and LD, was rejected. For LD, *cg* was similar to *c*, while *c-g* led to a decrease in the FS. This is in accordance with a previous report, where one-step glazing showed higher fatigue strength of LD than two-step [16]. However, for ALD, the result was the complete opposite. Both one- and two-step glazing improved its flexural strength compared to ALD*c*, whereas *c-g* was twice higher than ALD*c*. It is interesting that ALD material presented an initial strength that was only half of LD but showed the highest strength similar to LD after two-step glazing. So it is of great importance to understand how such glazing influences ALD mechanical properties in order to improve the mechanical performance of restorations. In addition, the influence of glazing on the flexural strength of these lithium disilicate ceramics cannot be explained by the change in surface roughness since the rougher surface could lead to lower strength [24].

The presence of a second fire cycle increased the strength of ALD. Additionally, if the second firing is for a glaze layer application, it improved even more of the material’s strength. However, only a second firing did not increase the strength of LD*c.* And the application of glaze in the second firing even decreased its strength. Therefore, the third hypothesis that refiring or the use of glaze material would not affect the strength of ALD and LD was partially rejected. This behavior can be justified because; during the second firing, the glassy phase on the glass ceramics could be partially molten at temperatures above the glass transition temperature (Tg). For the lithium disilicate reinforced glass ceramics, this temperature is around 450°C [25, 26]. With the temperature rising, the glass phase becomes less viscous to fill in and seal surface defects such as machining damages [27]. This also leads to a higher strength of ALD*c-g* compared to ALD*cg* since the first has an additional firing cycle. To verify this assumption, a crack healing analysis of ALD and LD was conducted in this study. As shown in Fig. 3, the introduced crack in ALD totally disappeared after the second firing. Therefore, surface damages caused by specimen preparation could be slightly healed in the first firing and further the subsequent firing, decreasing the critical defect size and therefore improving the ceramic strength. However, the crack in LD was only partially closed, and this change in LD failed to affect the average roughness and strength in this study, which can be supported by other studies that show a normal glazing cycle cannot improve the strength of LD [18]. It is possible to hypothesize that the glazing temperature of the two-step glazing might be insufficient for LD to influence the glassy phase of it as for ALD. An extended glaze firing with a longer holding time up to 15 minutes was suggested for LD to improve its mechanical properties [17, 18, 28]. In contrast, the strength improvement of ALD after an additional firing suggested that the initial firing by the manufacturer is insufficient for the material. Thus, longer firing time could be able to increase the strength of ALD as more firing cycles.

The application of glaze at different processing stages showed different effects on the flexural strength of ALD and LD. Comparing the FS of glazed and unglazed LD with one or two firings, the glaze material applied in the first firing did not generate an influence, while the glaze in the second firing decreased the FS. The first firing of LD could be sufficient for surface damage healing reaching its highest strength. This can be supported by the above findings that no polishing scratch was found in LD*c*, and all the critical defects are volume defects inside the ceramic. Different from LD*cg*, a number of large pores were detected under SEM at the interface between LD and the glaze layer, which can be the result of impurities induced before the second firing. These large pores act as the stress concentration while loading, weakening the mechanical properties of glazed restoration. Moreover, crystallized LD presented less glass percentage [4] and porosity than uncrystallized LD, and the holding temperature of the second firing was lower than the first firing. These conditions might influence the fusion of the glaze layer to LD substrate, therefore weakening the interface. For ALD, the application of glaze material at both the first and second firing leads to higher strength. It could be attributed that silicate glass, the major component of the glaze, potentially fills surface cracks during firing, collaborating with the crack-healing ability of ALD and compensating for the lack of firing. More evidence is needed to explain the effect of glaze on ALD strength.

In the study of Lubauer et al. [4], crystallized ALD presented a fracture toughness of 1.45 MPa·m^1/2^, which was relatively lower than crystallized LD (2.13 MPa · m^1/2^). This could partially explain that crystallized ALD without a glaze layer had a lower flexural strength than crystallized LD in the present investigation. However, despite sharing clinical indications [20], both ceramics present different performances, such as ALD demonstrating a better wear behavior than LD [29], and comparable bonding strength before and after aging [30]. Other investigations reported higher mean strength value (374.22 MPa) than this study (282.1 MPa) for ALD*cg* [31], which could be possibly explained due the difference between biaxial flexural strength and 3-point bending tests. In addition, the manufacturer claims that ALD flexural strength can achieve higher values than 700 MPa. In this study, even the strongest group (ALD*c-g*) presented 442.3 MPa, which is 63% of the value suggested by the manufacturer, but still superior to the threshold of 300 MPa defined in ISO 6872:2015.

As limitations of this study, in addition to the crack healing effect, it is unknown if the residual stress after glazing and refiring played a role in the different strength results. Also, the effect of successive thermal cycles can possibly lead to a change in crystal contents [16]. Repeated firings cause ceramic materials to be exposed to additional heat treatments. For nonreinforced glass-ceramics, the multiple firing did not affect the flexural strength, hardness, and microstructure in the long-term [32]. While for LD, the repeat firing processes did not affect its flexural strength, the surface hardness and fracture toughness were significantly changed [33]. However, there is a lack of information about that for ALD and low-temperature fused glass ceramics such as glaze. Thus, further studies are advocated to explore the crystal content after different glazing and refiring on the flexural strength of ALD and LD. Moreover, only two firing cycles were evaluated, so it is unknown if ALD can achieve higher strength with more firing cycles or longer firing time. The influence of the number and holding time of firing on the optical properties of ALD is still unknown. Further studies are suggested with a focus on the effect of firing cycles and longer holding time on the mechanical properties as well as the optical properties of ALD. In addition, laboratory studies evaluating fatigue and wear resistance would also improve the literature regarding this new lithium disilicate.

## Conclusion

Besides both materials being lithium-disilicate glass ceramics, the glazing technique and firing protocol affected their roughness and flexural strength differently. A two-step crystallization and glazing should be the first choice for ALD, while for LD, glazing is optional and, when necessary, should be applied in one step. Although glazing can generate a homogenous and glossy surface morphology, it increases the surface roughness for both materials.

## Data Availability

The data will be available when requested to the corresponding author.
